# A Case Report Emphasizing the Importance of Early Diagnosis and Management of Intracranial Germinoma

**DOI:** 10.7759/cureus.11721

**Published:** 2020-11-26

**Authors:** Nanik Ram, Sumera Batool, Naureen Mushtaq

**Affiliations:** 1 Internal Medicine: Diabetes and Endocrinology, Aga Khan University Hospital, Karachi, PAK; 2 Paediatric Oncology, Aga Khan University Hospital, Karachi, PAK

**Keywords:** bitemporal hemianopia, panhypopituitarism, tanner stage, germinoma, germ cell tumour

## Abstract

Intracranial germ cell tumors (GCTs) account for 3%-5% of all intracranial tumors. They commonly manifest during first two decades of life. We are reporting a case of a young female, who presented with progressive visual loss, polyuria and polydipsia, harboring an intracranial GCT. She presented initially to a neurosurgery clinic and then to an endocrine clinic, with a history of chronic worsening headache and recent onset visual blurring along with polyuria with polydipsia. On further inquiry, she was found to have primary amenorrhea, easy fatigability, and failure of development of secondary sexual characteristics. On examination the patient had bitemporal hemianopia with breast development at tanner stage II and pubic and axillary hair at tanner stage I. Her initial hormonal workup was suggestive of panhypopituitarism with diabetes insipidus. MRI pituitary showed a sellar mass with suprasellar extension, so an initial impression of a pituitary macroadenoma was made and the patient underwent trans-sphenoidal surgery. The histopathology was suggestive of lymphoid hyperplasia. Follow up MRI showed significant residual tumor and her vision and pituitary function did not recover. Neurosurgery was planned as second surgery, but we requested a second opinion of histopathology report and it was suggestive of a germinoma. She was then started on chemotherapy followed by radiotherapy, after which her tumor size reduced significantly, though she still required pituitary hormone replacement therapy.

Pituitary stalk lesions are rare and their diagnosis is challenging as different etiologies present clinically and radiologically in a similar manner with tissue diagnosis being the gold standard. Germinoma is a radiosensitive tumor. In our patient it took a long time to reach the correct diagnosis and late diagnosis resulted in permanent visual field defect and panhypopituitarism. This case report emphasizes that we should guide and educate our patients to seek medical advice early in the course of disease. We should also keep differential diagnosis in mind before referring the patient for surgery.

## Introduction

The central nervous system germ cell tumors (GCTs) are rare neoplasms [1]. They account for 3%-5% of all intracranial tumors but its prevalence varies. In Europe it is 0.1%-2.4% while in Japan and East Asia it varies between 2.1% and 9.5% [1-2]. These GCTs have a predilection for midline structures and commonly affect the pineal gland and suprasellar region in 50%-65% and 25%-35% of cases respectively [3]. The occurrence of GCTs at two different sites is referred to as bifocal GCTs and is very characteristic of these tumors [4].

Intracranial GCTs are divided into two groups, germinomas and nongerminomatous GCTs. Germinomas are more common accounting for two third of total cases [5].

We are reporting a case of intracranial GCT, which presented as a sellar and suprasellar mass and was initially diagnosed as nonfunctioning pituitary macroadenoma.

## Case presentation

A 21-year-old female presented to a neurosurgery clinic with worsening of headache for last three to four months. It started almost three years back and was gradual in onset and diffuse in nature. It was associated with blurring of vision especially on the right side, which started few days before presentation. There was no history of fever, loss of consciousness, or seizure. On further inquiry, it was revealed that she had history of polyuria and polydipsia since the age of 14 years. She never menstruated spontaneously and was using hormone (estrogen preparations) therapy intermittently. There was no history of galactorrhea.

On physical examination, she was vitally stable with a BMI of 25.2 (BMI for Asians - 18.5-23). Breast development was tanner stage II, while the axillary and pubic hair were tanner stage I. She had bitemporal hemianopia on visual field examination which was later confirmed on perimetry also. For workup, an MRI brain was performed three years ago which showed a mass arising from the pituitary stalk measuring 1.5 cm x 1.5 cm x 1.4 cm. She was advised surgery of that mass, for which she refused.

Now upon presentation to endocrine clinic, her hormone profile was performed as shown in Table 1. According to these results, a diagnosis of panhypopituitarism with diabetes insipidus was made.

**Table 1:** Results of hormone profile. FSH, follicle stimulating hormone; LH, luteinizing hormone; TSH, thyroid stimulating hormone; IGF-1, insulin-like growth factor

Test	Results	Normal ranges
FSH	1.54 mIU/mL	Follicular phase (1.4-9.9 mIU/mL)
LH	<0.07 mIU/mL	Follicular phase (1.7-15 mIU/mL)
Estradiol	22 pg/mL	Follicular phase (19.5-144.2 pg/mL)
TSH	2.02 uIU/mL	0.4-4.2 uIU/mL
FT4	0.41 ng/dL	0.89-1.76 ng/dL
9 am Cortisol	0.70 ug/dL	4.3-22.4 ug/dL
Prolactin	18.20 ng/mL	3.8-23 ng/mL
IGF-1	41.65 ng/mL	149.1-332.3 ng/mL
Fasting blood sugar	98 mg/dL	70-100 mg/dL
Urine osmolality	57 mosm/kg	50-1400 mosm/kg
Plasma osmolality	295 mosm/kg	275-300 mosm/kg
Serum sodium	146 mmol/L	136-145 mmol/L

**Figure 1:**
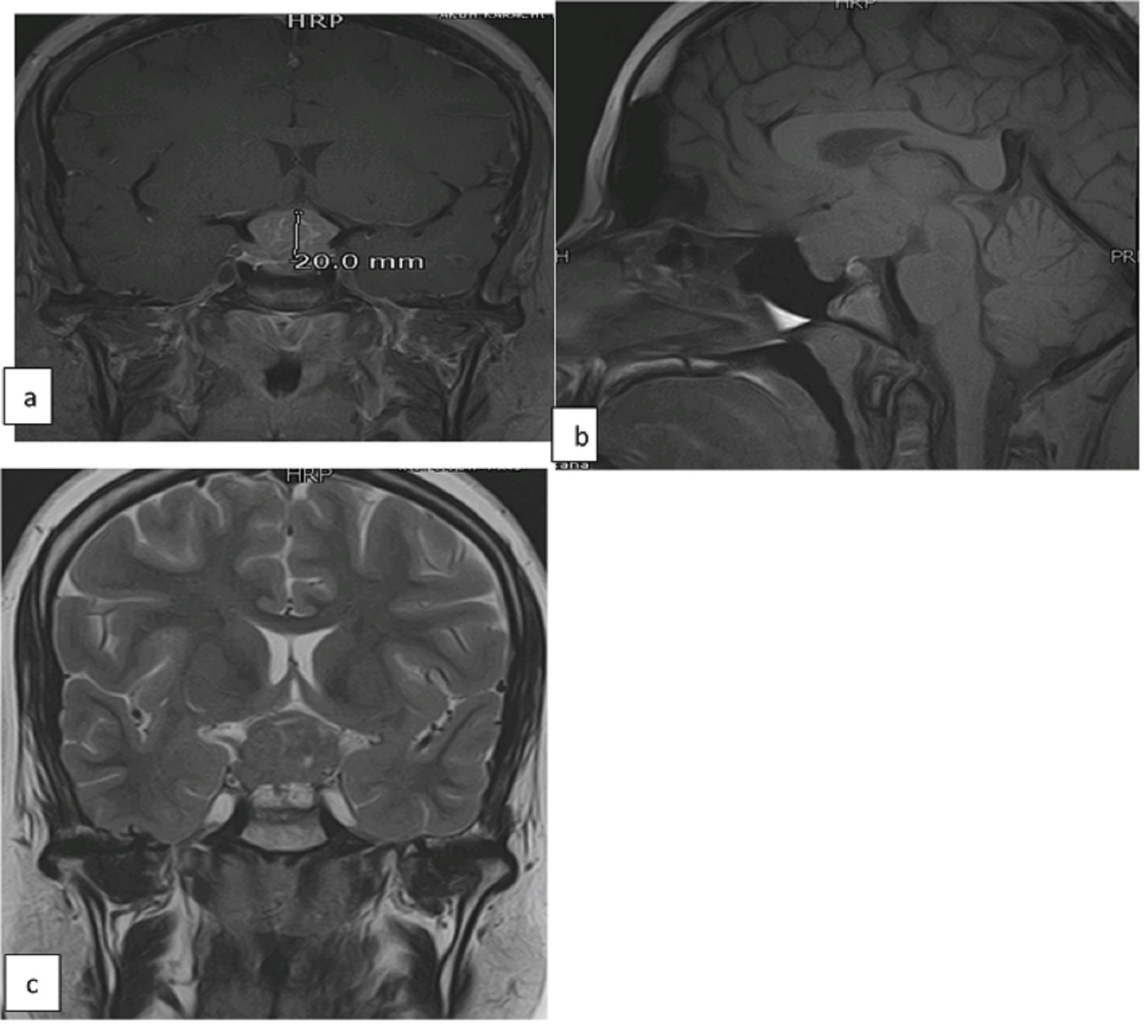
Preoperative MRI pituitary gland with (a) T1 coronal, (b) T1 sagittal, and (c) T2 coronal images showing a lobulated mass in the sellar region with suprasellar extension. It is iso to hyperintense on T1W image and isointense on T2W image.

She was started on hormone replacement therapy with thyroxine and hydrocortisone along with oral desmopressin tablets. A repeat MRI brain reported a sellar mass 3.3 cm x 2.4 cm x 2.2 cm which was extending to suprasellar region. Pituitary gland was inferiorly displaced and compressed by this mass and the two were inseparable from the left side. The optic chiasma was displaced superiorly (Figure 1).

This was considered as the nonfunctional pituitary macroadenoma. She underwent trans-sphenoidal resection of that mass. Her vision partially improved after surgery. To our surprise, the histopathology report was negative for pituitary adenoma; instead it showed reactive lymphoid hyperplasia and a diagnosis of lymphocytic hypophysitis was considered.

**Figure 2:**
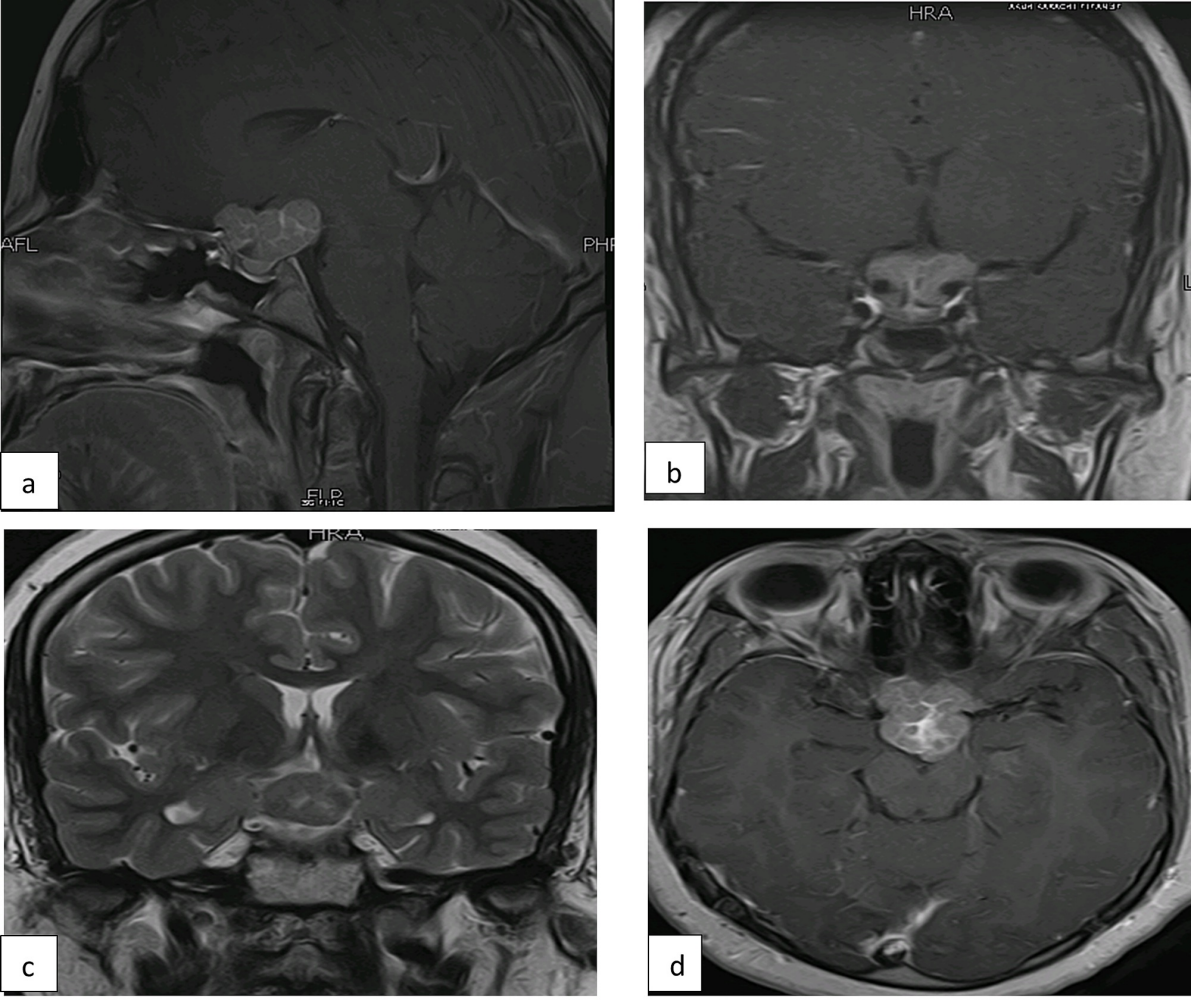
Follow-up MRI pituitary three months after surgery. It shows T1 postcontrast (a) sagittal, (b) coronal, (c) T2 coronal, and (d) axial images highlighting a heterogenous sellar mass with suprasellar extension with mild decrease in bulk overall. It is inseparable from the pituitary stalk and is displacing the optic chiasma superiorly.

She was continued on hormone replacement therapy and follow up MRI was done after three months of surgery. It redemonstrated a heterogeneous sellar mass with suprasellar extension which was mildly decreased in bulk overall with a size of 26 mm x 23 mm x 14 mm. However, the pituitary gland was separately visualized this time with interval resolution of mass effect (Figure 2).

**Figure 3:**
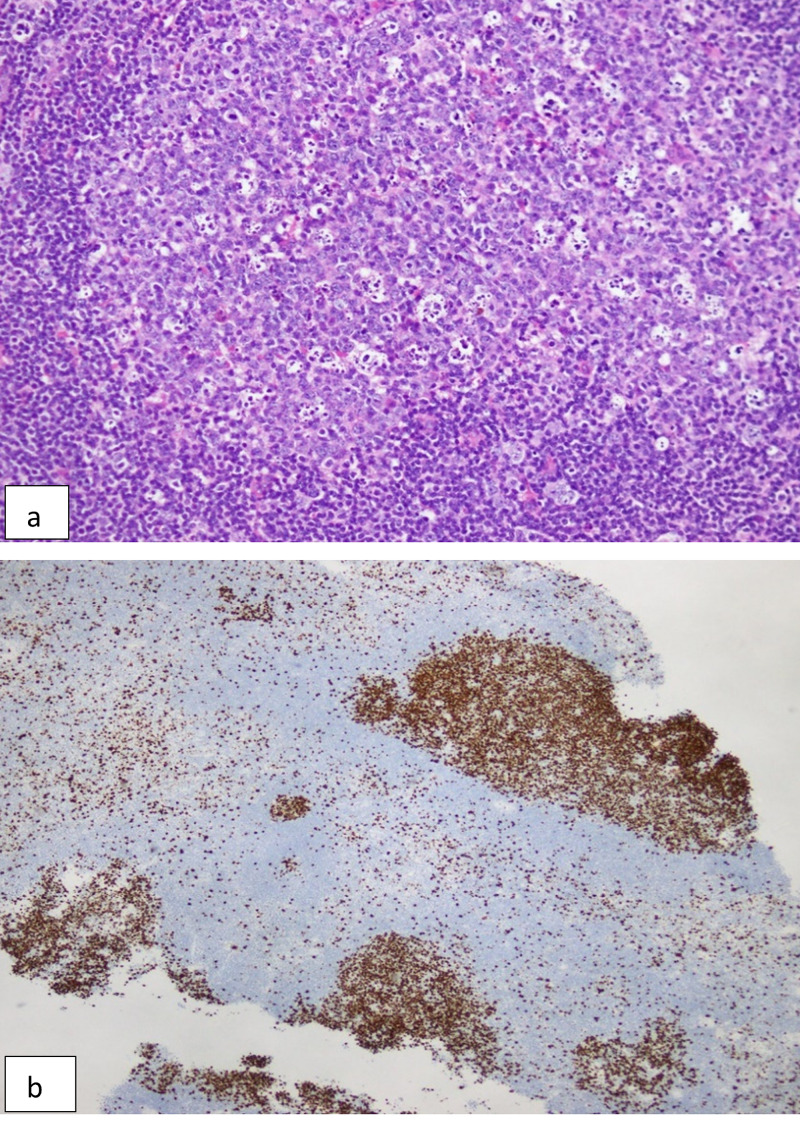
(a) Multiple fragments of lymphoid tissue present predominantly exhibiting variable sized lymphoid follicles. (b) Immunohistochemistry stains SALL-4, OCT-3/4 and CD117 highlight individually scattered germinomatous cells.

Her vision improved slightly after surgery. At this stage, it was keen to perform neurosurgeryas as a second surgery but before that, we decided to take a second opinion of histopathology. The slides were sent to a lab in Canada, where based on additional immunohistochemistry markers, a diagnosis of germinoma was made (Figure 3). These immunohistochemistry markers are not available in our country.

Her serum human chorionic gonadotropin (hCG) and alpha fetoprotein (AFP) were also normal but cerebrospinal fluid (CSF) could not be checked for these tumor markers due to nonavailability.

After discussion with oncologist and radiation oncologist, she was started on chemotherapy as per CARE protocol for the intracranial GCT. It included carboplatin 600 mg/m2 for day 1, etoposide 150 mg/m2 for days 1-3, and this course was repeated every three to four weeks. A total of four cycles were given followed by a radiotherapy boost 54 Gy including 24 Gy to the whole ventricle. Chemotherapy and radiotherapy were uneventful. A follow up MRI was done six months after radiotherapy which showed that the tumor size has decreased to 14 mm x 6 mm which can be a scar tissue only. However, her pituitary function did not recover and she still required thyroid and cortisol replacement along with desmopressin for her diabetes insipidus.

## Discussion

Most common intracranial GCT is germinoma and suprasellar mass is one of its common presenting features, as found in our patient. It arises from the primordial germ cells from the yolk sac endoderm that have aberrant migration to gonadal ridges during embryonic development and then later transformed into malignant cells [6]. It predominantly involves male gender and this particularly holds true for pineal gland germinomas [7]. The suprasellar germinoma is more common in females. The median age of diagnosis is 10-12 years of age and almost 90% of cases are diagnosed before the age of 20 years [8-9]. Our patient presented at the age of 21 years that made us think more in favor of pituitary macroadenoma rather than germinoma. However, she had symptoms present since the age of 14 years.

The clinical presentation varies according to the location and size of tumor [10]. The suprasellar mass affects the pituitary functions as our patient presented with panhypopituitarism. Another characteristic finding is the presence of central diabetes insipidus present in our patient as well [2]. Delayed diagnosis and treatment can further lead to obstructive hydrocephalous or spread to surrounding structures [9].

Our patient had hypogonadotropic hypogonadism with delayed puberty and primary amenorrhea. This points to the fact that the tumor had been present for quite some time and remained undiagnosed. Moreover, when it was found on MRI pituitary three years before presentation to us, the patient refused treatment for the fear of surgery.

For diagnosis, neuroimaging studies like MRI is sensitive for sellar and suprasellar lesions [11]. Our patient’s first MRI showed a mass arising from the pituitary stalk. Follow up MRI also suggested a huge sellar and suprasellar mass with an impression of a nonfunctioning pituitary macroadenoma. Till this stage she had developed visual symptoms, so she agreed for surgery. Radiological findings alone are not enough to distinguish germinoma from a nongerminomatous GCTs or other tumors like pituitary macroadenoma. Because of panhypopituitarism, bitemporal hemianopia and radiological features of sellar and suprasellar mass, we considered pituitary adenoma as the topmost differential diagnosis followed by any infiltrative lesion. Definite diagnosis in such cases requires histopathological confirmation. Serum and CSF markers like AFP and human chorionic gonadotrophin (hCG) can also help to differentiate the germinomatous and nongerminomatous tumors as pure germinomas are usually nonsecretory while nongerminomatous tumors present with elevated tumor markers [12]. Other than pituitary macroadenoma, an important differential diagnosis is lymphocytic hypophysitis, which also presents with stalk thickening and diabetes insipidus. In our case the initial biopsy report did show reactive lymphoid hyperplasia, and a diagnosis of lymphocytic hypophysitis was made.

However, a second opinion was sought before going for second surgery which confirmed the diagnosis of germinoma. Placental alkaline phosphatase (PLAP), c-kit, and OCT ¾ are very sensitive markers of germinomas [11]. Unfortunately, these markers are not available in our country. Being an immunogenic tumor, germinomas show extensive lymphocytic infiltration which might have led to the initial report of lymphoid hyperplasia in our patient. The absence of pituitary adenoma on the biopsy prompts us to look for other differential diagnosis. There are case reports in which the germinomas were initially misdiagnosed as lymphocytic hypophysitis [12]. 

Conventionally, surgery is considered first to obtain a tissue sample for biopsy followed by craniospinal irradiation. Surgical resection is also indicated when germinoma is complicated with obstructive hydrocephalous. Pure germinomas are very radiosensitive with a good overall prognosis. The five-year survival in germinoma patients who received radiotherapy was more than 88%-97% in one study [13]. The dose of radiation used in most cases was 50 Gy to primary tumor location and an extra prophylactic dose to craniospinal area. However, recurrence can occur after many years and craniospinal irradiation has long-term detrimental neurological sequels. So, treatment has been modified with the addition of chemotherapy along with the radiation, to reduce the dose of radiation and thus, reducing the risk of neurological complications in the long run. Chemotherapy alone has also been studied as a treatment modality but in a study in Taiwan, more than 50% patients had a recurrence within one to two years with this treatment option, so its use alone has not been recommended [14]. Thus, all germinoma patients first receive chemotherapy followed by cranial irradiation.

## Conclusions

Our patient had a long-time lag between the onset of symptoms and diagnosis with proper management. Initially the patient refused treatment due to fear of surgery and then was misdiagnosed as pituitary macroadenoma and lymphocytic hypophysitis. Till then she had developed irreversible visual damage and pituitary function loss. She still requires thyroxine, cyclic estrogen and progesterone therapy and hydrocortisone replacement along with desmopressin for diabetes insipidus.

This concludes that germinoma is a radiosensitive tumor with a favorable prognosis. But timely diagnosis and treatment is important. Presence of diabetes insipidus along with pituitary dysfunction should prompt the clinician to think differentials other than pituitary macroadenoma. Sellar and suprasellar lesions can mimic clinically and radiologically with pituitary macroadenoma or lymphocytic hypophysitis, so definite diagnosis of germinoma requires careful evaluation of histopathology.

## References

[1] Loto MG, Danilowicz K, González Abbati S, Torino R, Misiunas A (2014). Germinoma with involvement of midline and off-midline intracranial structures. Case Rep Endocrinol.

[2] Reddy MP, Saad AF, Doughty KE (2015). Intracranial germinoma. Proc (Bayl Univ Med Cent).

[3] (2018). Central nervous system germinoma: practice essentials, pathophysiology, etiology. https://emedicine.medscape.com/article/281714-overview.

[4] Phi JH, Kim S-K, Lee J (2013). The enigma of bifocal germ cell tumours in the suprasellar and pineal regions: synchronous lesions or metastasis?. J Neurosurg Pediatr.

[5] Jorsal T, Rørth M (2012). Intracranial germ cell tumours. A review with special reference to endocrine manifestations. Acta Oncol.

[6] Reisch N, Kühne-Eversmann L, Franke D (2009). Intracranial germinoma as a very rare cause of panhypopituitarism in a 23-year-old man. Exp Clin Endocrinol Diabetes.

[7] Takeuchi J, Handa H, Nagata I (1978). Suprasellar germinoma. J Neurosurg.

[8] Mufti ST, Jamal A (2012). Primary intracranial germ cell tumours. Asian J Neurosurg.

[9] Osorio DS, Allen JC (2015). Management of CNS germinoma. CNS Oncol.

[10] Packer RJ, Cohen BH, Cooney K (2000). Intracranial germ cell tumours. Oncologist.

[11] Suprasellar Germinoma: 2 Case Reports and Literature Review (2018). Suprasellar germinoma: 2 case reports and literature review. https://www.ncbi.nlm.nih.gov/pubmed/29913291.

[12] Ghorbani M, Moradi M, Ebrahim Khamseh M (2018). A case of pituitary germinoma misdiagnosed as lymphocytic hypophysitis. J Endocrinol Metab.

[13] González Bóssolo A, Mangual Garcia M, Davila K, Brau R, Sanchez Ortiz J, Hernan Martinez J (2018). A rare localized pituitary stalk germinoma presenting in the third decade. Case Rep Endocrinol.

[14] Chen Y-W, Huang P-I, Ho DM-T (2012). Change in treatment strategy for intracranial germinoma: long-term follow-up experience at a single institute. Cancer.

